# Prevalence and risk factors associated with nasal carriage of methicillin-resistant staphylococci in horses and their caregivers

**DOI:** 10.1186/s13567-024-01364-0

**Published:** 2024-09-09

**Authors:** Michela Bullone, Alessandro Bellato, Patrizia Robino, Patrizia Nebbia, Sara Morello, Daniela Marchis, Alberto Tarducci, Giuseppe Ru

**Affiliations:** 1 Department of Veterinary Sciences, University of Torino, Largo Paolo Braccini 2, 10095 Grugliasco, Italy; 2 Feed Hygiene Laboratory, Istituto Zooprofilattico Sperimentale del Piemonte, Liguria e Valle d’Aosta, via Bologna 148, 10154 Torino, Italy; 3 Biostatistics, Epidemiology and Risk Analysis Unit, Istituto Zooprofilattico Sperimentale del Piemonte, Liguria e Valle d’Aosta, via Bologna 220, 10154 Torino, Italy

**Keywords:** Horse, nasal carriage, methicillin-resistant staphylococci (MRS), risk factors, antimicrobial resistance, AMR genes

## Abstract

Antimicrobial resistance is a global threat, and pet-associated strains may pose a risk to human health. Equine veterinarians are at high risk of carrying methicillin-resistant staphylococci (MRS), but specific risk factors remain elusive, and few data are available for other personnel involved in the horse industry. The prevalence, characteristics, and risk factors for nasal carriage of MRS in horses and their caregivers were studied in northwestern Italy. Nasal swabs from 110 asymptomatic horses housed at 21 barns and 34 human caregivers were collected. Data on barns, horses, and personnel were acquired through questionnaires. The samples were incubated in selective media, and the bacterial isolates were identified by mass spectrometry. Risk factors were investigated by Poisson regression. MRS were isolated from 33 horses (30%), 11 humans (32.4%) and 3 environmental samples (14.2%). Most isolates were multidrug resistant (MDRS). The prevalence of MRS and MDRS was greater in racehorses and their personnel than in pleasurable and jumping/dressing horses. MRS carriage in caregivers was associated with an increased prevalence of MRS carriage in horses. The frequency of antimicrobial treatments administered in the barn during the last 12 months was a risk factor for MRS carriage in horses [prevalence ratio (PR) 3.97, 95% CI 1.11, 14.13] and caregivers (PR 2.00, 95% CI 1.05, 3.82), whereas a good ventilation index of the horse tabling environment was a protective factor (PR 0.43, 95% CI 0.20, 0.92). Our data reveal relevant interactions occurring between bacterial communities of horses and humans that share the same environment, suggesting that One Health surveillance programs should be implemented.

**Supplementary Information:**

The online version contains supplementary material available at 10.1186/s13567-024-01364-0.

## Introduction

Antimicrobial resistance represents a global threat to human and animal health. Livestock and pet-associated strains of antimicrobial-resistant staphylococci have been identified as possible, although uncommon, sources of infection in humans. Animal-associated *Staphylococcus* strains, both pathogens and commensals, can also act as reservoirs of antimicrobial resistance and virulence genes [1, 2]. From this perspective, veterinary epidemiological surveillance is a recognized fundamental process for the achievement of the One Health approach.

Among the resistance traits that staphylococci can acquire, methicillin resistance is considered the most important trait from a clinical point of view, as β-lactam antibiotics are the most prescribed class of antimicrobials in veterinary and human medicine [3, 4]. Methicillin resistance is mediated by the *mecA* or *mecC* genes, which encode alternative penicillin-binding proteins with a low affinity for β-lactam antibiotics. Methicillin-resistant *Staphylococcus aureus* (MRSA) is the bacterial agent of the *Staphylococcus* genus with the highest pathogenic potential, and it is recognized as a zoonosis [3, 5]. With increasing awareness of the global threat posed by antimicrobial resistance, surveillance for MRSA colonization in asymptomatic hosts has been implemented in many countries [6–9]. This made it possible to recognize equine veterinarians as being at increased risk of colonization or infection by MRSA compared with other veterinarians and non-veterinary people [10]. Less information is available concerning other methicillin-resistant staphylococci (MRS). Coagulase-negative staphylococci (CoNS) are mucosal and skin commensal bacteria characterized by a reduced capacity to cause acute, life-threatening infections compared with coagulase-positive staphylococci. CoNS and methicillin-resistant CoNS (MRCoNS) are, however, increasingly important pathogens that cause infections in immunocompromised human patients and after transplantation [11, 12]. The role of CoNS in equine infections is also increasingly recognized [13].

There is scientific evidence supporting the horizontal transmission of MRS strains between humans and horses (reviewed in [13]). A limited number of studies investigating the factors potentially associated with an increased prevalence of MRS colonization in asymptomatic horses and their caregivers are available [14–20]. The horses’ intended use or activity, namely, racing vs. other equestrian activities, as well as an increased number of horses stabled at the same facility, have been indicated as possible risk factors for MRCoNS and MRSA isolation in equine populations [16, 21–23]. Researchers have not explored whether horse caregivers are also subject to different risks on the basis of their horses’ intended use or activity.

The present study was designed to fulfil the following aims: (1) estimate the prevalence of nasal MRS colonization (in the manuscript from now on, described as MRS colonization) in asymptomatic horses stabled in our geographical area and their caregivers; (2) characterize the MRS strains isolated; and (3) assess the role of potential risk factors for MRS colonization in both species. On the basis of the literature mentioned above [16, 21–23], we hypothesized that racehorses and their caregivers are at increased risk of MRS colonization compared with pleasure horses and their caregivers.

## Materials and methods

The methodologies employed are fully detailed in Additional file 1.

### Study design

The study had a prospective cross-sectional design. Horse, personnel, and environmental swab samples were obtained from external barns (racing, riding or pleasure riding barns) and an internal teaching barn from our Department for MRS isolation and subsequent antimicrobial resistance profiling. The feed samples were obtained from external barns for antimicrobial residue testing. Specific questionnaires were employed to collect relevant information at the horse, personnel, and barn levels for risk factor analyses. The samples were collected from the end of July to the end of November 2019. Samples and data were collected by the same operators throughout the study period. Written informed consent was obtained from the owners of the studied horses and from the recruited people. The study was approved by the Animal Ethical and Welfare Committee of the Department of Veterinary Medicine (Prot. N. 936, 16/04/2019), University of Turin, and by the Ethical Committee of the Azienda Ospedaliera Universitaria San Luigi Gonzaga (Prot. 63/2019) for the equine and human parts, respectively. The procedures described for horses were performed in accordance with Directive 2010/63/EU on the protection of animals used for scientific purposes. The study conformed to the Declaration of Helsinki.

### External barns

Barns of Standardbred racehorses, show jumping or dressage horses (collectively described as show riding horses), and pleasure riding horses located in the Turin area were selected through convenience sampling and included a 1:1:1 ratio until reaching a total of 21 barns. Only barns with ≥ 7 horses were selected to increase the study power, on the basis of previous data indicating that the number of horses stabled at the barn is a risk factor for MRS colonization [16].

### Internal barn

The internal teaching barn of the Department of Veterinary Medicine of the University of Turin was included as a putative positive control barn, as a high prevalence of MRS was expected both in horses and humans working in this environment. It was located in close proximity to the equine hospital barn. Five asymptomatic horses permanently stabled at the teaching barn and an equine veterinarian working there were sampled, as they were expected to be at higher risk of MRS colonization on the basis of the available literature [10, 24, 25].

### Horses

For each external barn, four to seven horses were sampled, randomly chosen among those considered eligible. The inclusion criteria were (i) being clinically asymptomatic, as reported by the owner and confirmed by interviewing the attending veterinarian, and (ii) not having received any antimicrobial treatment in the previous two months. This was considered the shortest time required for an altered bacterial flora to recover from antimicrobial treatment on the basis of available evidence [15, 26]. Horses reluctant to have their nose manipulated were excluded a priori.

### Personnel

Personnel working at the barns studied were included on a voluntary basis, with a maximum of two persons per barn.

### Questionnaires and feed data collection

Data were collected by means of specific questionnaires (Additional file 2) at three levels: barn (same data for all horses sampled in that barn), horse (animal individual data), and personnel (human individual data). Further details on the gathered information are available online. Whenever possible, barn-level questions were asked to horse owners or to barn personnel not included in the study to validate the responses. Similarly, data concerning pharmacological treatments for horses were validated by interviewing the attending veterinarian. In the case of contrasting information, data were excluded from the analysis. Information concerning the feeds sampled was also obtained at the barn (name of the product, stocking modalities, and picture of commercial label when available) and by means of computer-based research (company producing feed for other animal species or medicated feed).

### Nasal sampling

Fifteen cm long rayan-tipped swabs (Transystem™ 110 C, Copan Diagnostics Inc.) moistened with sterile saline solution were employed. Swabs were inserted approximately 15 cm into both horse nares and 2 cm into one nostril, which was randomly chosen, of each person studied. The swabs were gently pushed and rotated onto the equine or human nasal mucosa for a minimum of 5 s, trying to sample the whole mucosal area down to the external naris openings. In horses, care was taken to introduce the swab deep in the ventral meatus and to sample the mucosa of the nasal vestibulum while moving rostrally, as this site was associated with increased sensitivity for MRSA carrier identification in horses [27]. The same swab was used to sample both nostrils of the same horse. The swabs were discarded, and the procedure was repeated if contamination occurred during sampling.

### Environmental sampling

One environmental sample per barn was obtained using a 15 cm long rayan-tipped swab over a 10 cm^2^ area, typically from a lateral wall of the shower area. This sampling site was chosen because the shower area is used by all horses from the stable.

### MRS isolation and assessment

The swabs were kept at 4 °C in Amies agar gel transport medium and processed within 24 h for selective isolation of MRS spp. following standard protocols [28, 29]. Isolated colonies were initially identified by morphology, Gram staining, culture media colour changes, and catalase and coagulase tests. Bacterial species identification was performed via whole-cell MALDI-TOF mass spectrometry (Microflex^®^, Bruker Daltonics Inc.). DNA was extracted from the isolates, and PCR was performed for the 16 S, *mecA*, *mecC* and *nuc* genes as previously described [30].

Antimicrobial susceptibilities of the isolated colonies were determined using the Kirby–Bauer disk-diffusion method for penicillin, ceftiofur Na, gentamicin, enrofloxacin, tetracycline, erythromycin, and trimethoprim-sulfamethoxazole. EUCAST breakpoints were used to define resistance, except for ceftiofur and enrofloxacin, for which they were unavailable. CLSI breakpoints have been used instead. *Staphylococcus* spp. resistant to three or more antimicrobial molecules among those tested were defined as multidrug-resistant (MDR) staphylococci (MDRS).

### Feed sampling and assessment

Feed samples of approximately 500 g each were collected from feed storage bins or tanks, placed in triple plastic bags, and stored at −20 °C until subsequent analysis. Only the feeds consumed by the horses sampled were selected in a variable number on the basis of their diets, with no restrictions. Hay was not sampled. Antimicrobial residue analysis of the feed was performed via multiresidue UPLC‒MS/MS, which allowed the detection of 43 target antibiotics (Additional file 3) with a limit of detection (LOD) of 50 µg/kg and a limit of quantification (LOQ) of 250 µg/kg.

### Statistical analysis

Data from the internal barn are described but are consistently excluded from the analyses. In the evolving panorama of AMR in veterinary medicine, the inclusion of this barn served to confirm observations reported in previous data and the appropriateness of any comparison with previous literature. Data analysis was performed via STATA v15.1 (StataCorp LLC, College Station, Texas, USA). Continuous data are expressed as the mean ± standard deviation (SD) and were compared with the Kruskal‒Wallis test, as all datasets assessed were not normally distributed according to the Shapiro‒Wilk normality test. The chi-square test was used to compare data expressed as proportions. The prevalence of MDRS colonization was used to obtain prevalence ratios (PRs) and relative 95% CIs via a robust variance estimate. The frequencies of travel and antimicrobial treatments were calculated as rates and expressed as events/horse-months. Using pleasure riding horses as a reference category, the travel rate ratio and antimicrobial treatment rate ratio were obtained using Poisson regression with robust cluster variance estimates.

Risk factors for nasal colonization by ≥ 1 MRS (dichotomous dependent variable) were initially assessed with bivariate unadjusted Poisson regression with the alpha set at 0.1. Variables perfectly associated with the outcome at this step were excluded from further analyses, as the observed effect was attributed to the low number of observations available. As our questionnaire included multiple variables concerning air quality (number of windows or openings in the box, paddock exposure, and number of hours at paddock per day), considering the importance attributed to the airborne transmission of respiratory pathogens, an ordinary variable named the ventilation index was created and included in the risk analysis. The ventilation index was attributed to a value of 0, or poor, for horses stabled in a box with only one opening for ≥ 22 h/day (weekly mean; which corresponds to ≤ 2 h/day at pasture); a value of 1, or good, for horses stabled in a box with only one opening for ≥ 12 and ˂22 h per day (weekly mean; corresponding to > 2 and up to 12 h/day at pasture), or stabled in a box with > 1 opening, independent of turnout time; and a value of 2, or optimal, for horses kept at pasture for > 12 h per day (weekly mean), independent of the number of box openings. A minimum size of 80 × 80 cm^2^ was used to define box openings. Boxes with one or more lateral walls not reaching the roof were considered to have ˃1 opening. Variables found to be associated with the outcome were included in mixed-effect Poisson multivariate analyses where the barn was treated as a within-subject (random) factor. Multicollinearity was expected and assessed by uncentered variance inflation factors (VIFs). If ≥ 2.5, the covariates were singularly removed from the model to prevent multicollinearity-induced bias [31], and the model was repeated until the VIFs of all the covariates were < 2.5. Variance estimates were always adjusted for clusters (barn). The goodness of fit of the Poisson regression models used was tested via Pearson and deviance statistics.

## Results

Twenty-one external barns were studied: six Standardbred racing barns, seven show riding, and eight pleasure riding barns. The details are reported in Table 1.

**Table 1:** **Details of the barns and horses studied**

	Internal barn	Racehorse barns	Show riding barns	Pleasure riding barns	All external barns
Barns [n]	1	6	7	8	21
Barns where tacks are shared [n]	1	4	2	3	9
Total horses [n]	5	104	225	286	615
Horses per barn [n]	5	17 ± 9	32 ± 15	32 ± 14	27 ± 14
Total horses sampled [n]	5	33	38	39	110
Age [years]	16.0 ± 7.4	5.5 ± 3.6	13.0 ± 4.4*	13.6 ± 6.8*	11.1 ± 6.3
Sex [M: F]	2:3	17:14	25:13	23:16	65:43
Weight [kg]	476 ± 33	440 ± 52	516 ± 93*	478 ± 170	481 ± 123
Travelling rate ratio [IRR (95%CI)]	–	3.06 (1.13, 8.27)	1.48 (0.49, 4.51)	1.00	–
Hospitalizations last year [n]	0	1	4	0	5
Horses intended for food consumption [n]	1	3	0	7	10
Horse-specific antimicrobial treatment rate ratio [IRR (95% CI)]	–	3.16 (0.84, 11.84)	2.31 (0.63, 8.54)	1.00	–
Barn-estimated antimicrobial treatment rate ratio [IRR (95% CI)]	1.50	10.00 (4.16, 23.97)	2.00 (0.81, 4.97)	1.00	–

The data are expressed as absolute counts or means ± SD. Data from the internal barn were systemically excluded from the statistical analyses. “All external barns” includes data from the 21 racehorses and shows riding barns. Barn estimates of antimicrobial treatments administered are based on personnel interviews and cover all horses stabled at the barn during the 12 months preceding the interview. Horse-specific values are based on personnel interviews and cover antimicrobial treatments administered in the last 12 months only to the horses included in the study. IRR were compared with Poisson regression and robust variance estimates.

*Significantly different from racehorse barns (*p* < 0.05, Kruskal‒Wallis test).

### Horses

The horses from the external barns (*n* = 110) included 32 Standardbred racehorses, 13 ponies, 5 draft horses, 51 show jumping horses (French, Italian, German, or mixed breed), 5 Quarter horse-related breeds, and 4 Lipizzaner horses. Information on pharmacological treatment was always in agreement when verified; thus, there was no exclusion from the database due to data disagreement. Racehorses (*n* = 33) were younger compared to show (*n* = 38) and pleasure riding (*n* = 39) horses and had shorter permanence times (length of stay) at the barn compared to pleasure horses (Table 1). The rates of travel and barn-estimated antimicrobial treatment administration were higher in racehorses than in pleasure horses. After bacterial species identification, 33 isolates (from 33 horses out of 110) were recognized as MRS, corresponding to an overall 30% prevalence of MRS colonization in asymptomatic horses from our region (95% CI 21.6, 39.5%). In the same population, the prevalence of MDRS colonization was 18.2% (95% CI 11.4, 27.7%). Details of the nonstaphylococcal methicillin-resistant isolates are described in Additional file 1. The prevalence of MRS and MDRS colonization was higher in racehorses than in pleasure horses (Table 2). The horse-associated MRS were all MRCoNS, mainly *S. sciuri* (*n* = 17) and *S. fleurettii* (*n* = 9), with lower prevalences of *S. equorum* (*n* = 3), *S. lentus* (*n* = 2), *S. cohnii* (*n* = 1) and *S. saprophyticus* (*n* = 1). All the isolates expressed the 16 S gene, 26 expressed the *mecA* gene, and two expressed both the *mecA* and the *mecC* genes. None of them expressed the *nuc* gene. Among the 33 MRS, nine were resistant to one class of antimicrobial, five were resistant to two classes of antimicrobial, and 20 were MDRS (60.6%, 95% CI 42.1, 77.1%). In the internal barn, eight MRS were isolated from the five horses sampled, corresponding to a 100% prevalence. They were identified as *S. aureus* (*n* = 1), *S. equorum* (*n* = 1), *S. fleurettii* (*n* = 2), *S. lentus* (*n* = 2) and *S. sciuri* (*n* = 2). One horse harboured four isolates. Seven isolates expressed the *mecA* gene; one expressed the *nuc* gene (*S. aureus*), whereas none expressed the *mecC* gene. Among the five isolates from the teaching herd, one showed in vitro resistance to one class of antimicrobial, two to two classes of antimicrobial, and five to three or more antimicrobial agents (MDRS).

**Table 2:** **Nasal carriage of MRS and MDRS in horses by their intended use**

	Internal barn	Racehorses	Show horses	Pleasure horses	All external barns
Horses [n]	5	33	38	39	110
Horses with ≥ 1 MRS [n]	5	14	10	9	33
Prevalence of MRS carriage (95% CI)	1	0.42 (0.28, 0.58)	0.26 (0.13, 0.46)	0.23 (0.10, 0.45)	0.30 (0.21, 0.41)
Prevalence ratio for MRS carriage [PR (95% CI)]	–	1.84 (0.83, 4.07)	1.14 (0.44, 2.92)	1	–
Horses with ≥ 1 MDRS [n]	2	10	8	2	20
Prevalence of MDRS carriage (95% CI)	0.40	0.30 (0.16, 0.50)	0.21 (0.08, 0.44)	0.05 (0.01, 0.17)	0.18 (0.10, 0.30)
Prevalence ratio for MDRS carriage [PR (95% CI)]	–	5.91 (1.55, 22.52)	4.10 (0.96, 17.58)	1	–

“All external barns” includes data from the 21 racehorses and shows riding barns.

### Personnel

The personnel tested included 34 people taking care of the horses sampled in the external barns studied and one veterinarian working at the Equine University Teaching Hospital and internal barn. Details are provided in Table 3. The prevalence of MRS colonization was higher in personnel working with racehorses compared to show riding and pleasure horses, and a similar pattern was observed for MDRS (Table 4). MRS were identified in 11 human samples, corresponding to a 32% prevalence of MRS colonization (95% CI 17.4, 50.5%). *S. epidermidis* (*n* = 4), *S. sciuri* (*n* = 3), *S. cohnii* (*n* = 2), *S. hominis* (*n* = 1) and *S. haemolyticus* (*n* = 1) were identified, all MRCoNS. Two of these isolates were resistant to molecules from a single antimicrobial class, three were resistant to molecules from two antimicrobial classes, and six were MDR strains. Nine isolates expressed the *mecA* gene, whereas none of them expressed the *nuc* or *mecC* gene. The isolate obtained from the veterinarian was an MDR *S. aureus* strain expressing *mecA* and *nuc* genes that was resistant to all the antimicrobial molecules tested.

**Table 3:** **Details of the personnel studied**

	Internal barn	Racehorse barns	Show riding barns	Pleasure riding barns	All external barns
Human subjects [n]	1	11	10	13	34
Age [years]	45	45 ± 11	41 ± 13	32 ± 14	38 ± 13
Sex [M: F]	1:0	9:2	3:7	7:6	19:15
Time from recruitment [years]	18	12 ± 11	5 ± 4	11 ± 9	10 ± 9
Any disease [n]	0	1	1	2	4
Antimicrobial treatments in the last 2 months [n]	0	0	2	3	5
Antimicrobial treatment rate in the last 2 months [treatment/person-months]	0	0	0.10	0.11	0.07
Time spent at the barn daily [h	4	8 ± 2	12 ± 6	8 ± 4	9 ± 4
Time spent at the barn manipulating horses [h]	1	5 ± 2	7 ± 4	5 ± 3	6 ± 3
Hand washing daily frequency [n]	20	7 ± 6	12 ± 9	5 ± 5	7 ± 7

After excluding data from the internal barn, Kruskal-Wallis comparisons were run without detecting any statistically significant differences. “All external barns” includes data from the 21 racehorses and shows riding barns. The data are expressed as absolute counts or means ± SD.

**Table 4:** **Nasal carriage of MRS and MDRS for the personnel studied by horse intended use**

	Internal barn	Racehorse barns	Show riding barns	Pleasure riding barns	All external barns
Human subjects [n]	1	11	10	13	34
Human subjects with MRS [n]	1	8	1	2	11
Prevalence of human nasal MRS carriage (95% CI)	1.00 (1.00–1.00)	0.73 (0.31, 0.94)	0.10 (0.01, 0.48)	0.15 (0.03, 0.48)	0.32 (0.16–0.55)
Prevalence ratio for human nasal MRS carriage [PR (95% CI)]	–	4.72 (1.21, 18.40)	0.65 (0.07, 5.80)	1.00	–
Human subjects with MDRS [n]	1	5	0	1	6
Prevalence of human nasal MDRS carriage (95% CI)	1.00 (1.00, 1.00)	0.45 (0.17, 0.77)	0.00 (0.00, 0.00)	0.077 (0.01, 0.41)	0.18 (0.07–0.38)
Prevalence ratio for human nasal MDRS carriage [PR (95% CI)]	–	5.91 (0.81, 43.11)	0.00 (0.00, 0.00)	1.00	–

“All external barns” includes data from the 21 racehorses and shows riding barns.

### Environment

The environmental samples yielded a total of four MRS isolates from three barns (14.3%). They were all MRCoNS identified as *S. sciuri* (*n* = 2), *S. lentus* (*n* = 1) or *S. cohnii* (*n* = 1). The *mecA* gene was expressed by three (75%) MRS isolates. One MDR *S. equorum* strain expressing *mecA* was isolated from an environmental sample from a university barn.

### Antimicrobial residues in feeds

Twenty-one complimentary preparations and 13 single cereal feed samples (total *n* = 34) were collected from the 21 external barns studied (median 1; range 0–5). None of the tested antimicrobial molecules appeared to exceed the LOQ in the feeds studied. Traces of oxytetracycline, amoxicillin, sulfadimetoxin, and tiamulin were found in three of the 21 complementary feed samples, which were obtained from three different barns and provided by two of the 18 different manufacturers tested, both of which produced medicated feeds intended for livestock species.

### Risk factor analyses

The bivariate unadjusted analyses revealed barn activity, the habit of using shared tacks, the barn-estimated antimicrobial treatment ratio, being stabled in a box with > 1 window or opening, and the ventilation index as factors significantly associated with MRS colonization in horses (Additional file 1A). There were 25 horses kept exclusively at paddock. For them, information on the number of openings in their box was lacking. We choose to use the variable ventilation index instead of the number of openings in the box for inclusion in the multivariate model so that all 110 horses can be studied together. Additionally, barn activity was removed because it was considered biologically irrelevant. As fomites are a recognized transmission factor for respiratory infections in horses [32, 33], a significant association was expected and observed between shared tacks and the barn-estimated antimicrobial treatment ratio, with the latter being significantly higher in the presence vs. absence of shared tacks (mean ± SD, 0.58 ± 0.64, *n* = 45 vs. 0.18 ± 0.19, *n* = 65). Additionally, stratified analysis revealed different effects of the barn-estimated antimicrobial treatment ratio on MRS carriage in the presence and absence of shared tacks (PR 1.12, 95% CI 0.7, 1.75 vs. 4.44, 95% CI 1.22, 16.08). Given the potential implications of both variables as risk factors for MRS colonization, a further interaction factor was inserted into the model. The barn-estimated antimicrobial treatment ratio was recognized as a risk factor for MRS colonization in horses, with a significant interaction with shared tacks, while good and optimal ventilation indexes were protective factors compared with poor ventilation index (Table 5). The use of shared tacks per se was not recognized as a risk factor in our study, possibly due to a lack of power. The overall performance of the fitted model was good, as the Pearson and deviance statistics revealed a nonsignificant lack of fit (*p* = 0.99 and *p* = 0.98, respectively). When the ventilation index was modelled as a continuous variable instead of an ordinal variable, the statistical significance of the effect was maintained.

**Table 5:** **Mixed-effects multivariate Poisson analysis with barn included as a random effect and cluster variance (robust) estimates**

	PR	95% CI
Ventilation index		
Score 0	Ref.	–
Score 1	**0.43**	**0.20, 0.92**
Score 2	**0.44**	**0.20, 0.95**
Shared tacks	2.19	0.96, 4.99
Barn-estimated antimicrobial treatment ratio	**4.16**	**1.15, 15.01**
Interaction Shared tacks*Barn estimated antimicrobial treatment ratio	**0.22**	**0.05, 0.93**

The results are expressed as prevalence ratios (PR) for a horse being a nasal carrier of ≥ 1 MRS. *n* = 110. Bold indicates variables significantly associated with equine MRS carriage.

Bivariate analyses performed on personnel data revealed gender, barn activity (reference: pleasure riding), number of horses studied at the barn carrying nasal MRS, barn-estimated antimicrobial treatment ratio, and having a colleague (other personnel employed at the barn and participating in the study) carrying nasal MRS as potential risk factors associated with MRS colonization in horse caregivers (Additional file 1B). Again, barn activity was removed from the model. The final model identified the barn-estimated antimicrobial treatment ratio as the only risk factor significantly associated with human MRS colonization (Table 6). The overall performance of the fitted model was good, as determined by the Pearson and deviance statistics (*p* = 0.98 and *p* = 0.93, respectively).

**Table 6:** **Mixed-effects multivariate Poisson analysis with barn included as a random effect and robust cluster variance estimates**

	PR	95% CI
Sex [ref: female]	2.17	0.65, 7.21
Barn-estimated antimicrobial treatment ratio	**2.00**	**1.05, 3.82**
N horses carrying MRS	1.29	0.87, 1.92
Colleague carrying intranasal MRS	2.10	0.81, 5.49

The results are expressed as prevalence ratios (PR) for a human being who is a nasal carrier of ≥ 1 MRS. *n* = 34. Bold indicates variables significantly associated with human MRS carriage.

There were seven out of 21 external barns where MRS were isolated from nasal swabs of both horses and personnel. Among them, four were barns of racehorses (4/6, 67%), one was a show horse (1/7, 14%), and two were pleasure horses (2/8, 25%). The same bacterial species (*S. sciuri*) with the same antimicrobial resistance pattern was identified in both horses (*n* = 2 out of five tested) and personnel (*n* = 1 out of two tested), suggesting possible direct horizontal transmission. In two other barns, overlapping antimicrobial resistance patterns could be observed in human and equine isolates, but the bacterial species differed (*S. epidermidis* and *S. sciuri* in humans vs. *S. fleurettii* and *S. cohnii* in horses).

## Discussion

In the geographical area studied in northwestern Italy, MRS colonization was detected in one-third of the horses and personnel studied. The overall prevalence of MR-CoNS colonization observed in horses was consistent with the literature [6, 17, 18]. Our inability to detect MRSA was also in line with reported prevalence rates < 2% in horses in our region and, more broadly, in Europe and Canada [21, 23, 24, 34, 35]. Our sampling could not reveal the presence of MRSA colonization in the equine and human populations studied, with the only exception being one equine veterinarian working at our University Hospital. This finding also agrees with previous data indicating that equine veterinarians are at increased risk of nasal carriage of MRSA compared with people not professionally exposed to animals or to asymptomatic persons in the community [7–9]. More than half of the isolates were MDRS, with similar distributions across equine and human species. The results of this study indicate that antimicrobial treatment frequency in horses is the main factor associated with both equine and human nasal carriage of MRS. These findings emphasize the interactions among the bacterial communities of horses and humans in the same environment. Another finding of this study concerns the protective effect of better air quality against MRS carriage in the horses studied, supporting the well-recognized importance of good air exchange in controlling microbial spread in the community.

There is no doubt that the recent administration of antimicrobials can lead to resistance in bacterial populations of living organisms receiving treatment, particularly when they are used inappropriately. Published evidence shows that the administration of antimicrobial treatment during the last 30 days is a risk factor for MRSA nasal carriage in horses [15]. Much less is known concerning the dynamics of AMR acquisition by bacteria as well as the duration of colonization by MRS of the equine nose, although colonization times of up to 5 months have been reported. Despite the increased availability of data on the prevalence of MRSA and MRS in equine populations and equine industry personnel [36–39], studies of risk factors specifically associated with MRS carriage or colonization in horses and their caregivers remain scarce and limited to MRSA [20, 40]. From this perspective, our study was designed to specifically investigate risk factors other than recent antimicrobial treatment for MRS colonization of horses and their caregivers. In particular, variables used to study antimicrobial exposure in horses have been chosen to address the long-term impact of the total burden of antimicrobial treatments performed at the barn rather than in a single animal (barn-estimated antimicrobial treatment vs. horse-specific antimicrobial treatment in the last 12 months, indirect vs. direct effect/exposure). The inclusion criterion was to minimize the effect of recent direct exposure to antimicrobial treatments in the horses tested (e.g., to be free from antimicrobial treatment for at least 2 months before enrolment and sampling). The time interval of two months was chosen on the basis of data on AMR clearance times reported in non-staphylococcal species colonizing the equine gut [26]. The other variables studied as possible risk factors for horses included environmental contamination (from barn walls/fences or feeds with antimicrobial residues), the use of shared fomites in the barn, ventilation/air quality-related parameters, the frequency of travel, the number of horses stabled at the barns, and previous hospitalizations. Barn activity was also studied as a risk factor on the basis of previous work suggesting that racehorses are at increased risk of nasal MRS colonization compared with broodmares and riding horses [6]. It is hard to believe, however, that racing per se could be a risk factor for that. Rather, it is plausible that management practices or physical features of racehorses represent real risk factors for the reported increase in MRS prevalence. Race activity was associated with a younger age, frequent habit of sharing tacks, increased travel frequency, and increased frequency of antimicrobial treatments in our study. This latter point is also likely linked to the young age of the horses [41], and available data suggest that antimicrobials may be overused in racehorses [42]. In line with this, our model for predictors of equine MRS colonization had a high mean VIF when barn activity was included (collinearity), and removal of this variable resulted in an overall improvement in the VIF to values close to 1. The final analysis revealed barn-estimated antimicrobial treatments as the only significant risk factor for MRS carriage in horses, significantly interacting with the habit of sharing tacks, whereas good and optimal ventilation indexes appeared to be a significant protective factor in this regard. These findings confirm that antimicrobial administration represents a crucial determinant of antimicrobial resistance in commensal bacteria. The same findings also highlight the detrimental impact of indirect exposure to antimicrobial treatments.

The significant protective effect of variable ventilation on equine MRS colonization deserves attention. *Staphylococcus* spp. are recognized as microorganisms capable of airborne dissemination and transmission, even if the precise dynamics of airborne infection remain elusive [43]. The airborne spread of antimicrobial resistance genes and bacteria of animal origin into the environment and the colonization of humans who share the same environment have been recently described [44–46]. From this perspective, the so-called “open air factor”, wherein properties of outdoor air can reduce the viability and virulence of airborne microorganisms [40], might have played a role by decreasing humidity or increasing sunlight exposure of the environment in which the horses lived during our study. Our data suggest that, even for horses kept inside the barn for most of the day, good air quality (resulting from barn and box air exchange, overall air quality) may significantly contrast the ability of MRS to spread. The statistical significance of the test for the trend for the variable ventilation further suggests that at increasing levels of perceived air quality, there is a greater effect in terms of MRS carriage prevalence reduction. This also offers a rough validation of our air quality assessment method. The lack of a significant association between MRS carriage and variables concerning air quality other than the number of windows in the box where horses were housed at the initial regression analysis was likely due to the low power of the study. Although our work was not designed to study in detail the effects of the environment on bacterial loads or survival, our results support the paramount importance of adequate barn ventilation and turnout time in the management and wellbeing of horses and, indirectly, for people working with them.

For human MRS colonization, the risk factors studied were mainly related to horse management practices, the health conditions of the people studied, and hand hygiene. Our data support the fact that uncontrolled management practices in horses, especially concerning antimicrobial treatments, may act as an indirect risk factor for increased MRS colonization in caregivers and that the risk is increased under specific conditions, such as in racehorse barns. To the authors’ knowledge, there are currently no reports of the horizontal transfer of MR-CoNS between horses and humans. The increased prevalence of equine MRS isolates at the barn level associated with MRS colonization in personnel has also been reported for MRSA [3], suggesting that different *Staphylococcus spp*. may behave similarly concerning the mechanisms of acquisition of antimicrobial resistance genes. This, combined with the high prevalence of methicillin-sensitive *S. aureus* physiologically colonizing equine [47] and human nostrils and skin [48], should warn against the ease with which resistance genes could be transferred from CoNS to *S. aureus* or other coagulase-positive *Staphylococci* [49, 50], with important health implications.

The most common isolates from horses were *S. sciuri* and *S. fleurettii*. They were all MRCoNS. *S. sciuri* is commonly reported as a nose commensal in horses [15, 17, 19]. *S. fleurettii* was first isolated and described in 2000 from goat milk cheeses [20], and it is recognized as a component of the commensal flora of goats, pigs and small mammals [2]. *S. fleurettii* is intrinsically resistant to novobiocin [20]. The available literature suggests that *S. fleurettii* is a rare commensal of the horse nasal flora that is generally susceptible to methicillin [19]. This study is the first to report methicillin-resistant *S. fleurettii* colonization in horses, which was identified in 9 horses from 6 barns, and 7 out of 9 isolates were MDRS. All the isolates expressed the *mecA* gene, except for *S. cohnii*, *S. saprophyticus*, and 2 of the 17 *S. sciuri* isolates, which, however, presented phenotypic resistance to cefoxitine, penicillin, and/or ceftiofur. This may be because several homologues exist for the *mecA* gene, especially in MRCoNS [2], including those described in *S. sciuri* (*mecA1*) and *S. saprophyticus* (*mecC2*), with 79 and 92% identity with *mecA*, respectively. While previous evidence suggests that MRCoNS may play a role in the emergence of MRSA as a potential source and reservoir of the *mecA* gene, recent evidence challenges this unidirectional view, suggesting that it can also act as a protective factor against massive MRSA colonization in certain subsets of patients.

The data presented here were collected in 2019 after several warnings of antimicrobial disuse in animal species. At that time, regulations on antimicrobial residues in feeds (DGSAF 0021392-P-11/05/2015) only regarded animals intended for food consumption and were fixed at the maximal threshold of 1 mg/kg for all molecules except for penicillins, for which the limit was 0.5 mg/kg. Since then, however, research on this topic has shown that levels as low as 2 µg/kg trimethoprim can drive AMR in commensal bacteria from exposed horses, while insufficient data have prevented the drawing of relevant conclusions for β-lactams [51, 52]. These concentrations are very close to, and in some cases even lower than, the lower LOD of the available instruments. As most horses used for sports activities are not intended for food consumption, there are actually no regulations or controls on their feeds. In the context of the feed industry, medicated and nonmedicated feeds produced for different animal species can be produced from the same plants, using the same production line, following cleaning operations, which may be partly ineffective and result in unintended antimicrobial residue carry-over in feeds [53]. Given the relevance of horses as potential sources of AMR bacteria that can colonize humans, surveillance on this side may provide new meaningful information on AMR-driving mechanisms. Additionally, to further dissect the roles that several factors are likely to play in AMR development in the equine bacterial community, it must be acknowledged that, in 2019, Italy introduced a compulsory veterinary electronic prescription system. With this system, antimicrobial prescription was strictly regulated and controlled, resulting in a significant drop in annual prescription sales, as reported by the most recent ESVAC report [54].

In conclusion, our data confirm and expand previous findings in support of antimicrobial treatment exposure, either directly or indirectly, as the main determinant of MRS colonization in horses. Our findings highlight barn-level antimicrobial exposure as a risk factor for MRS colonization within an equine population, as well as evidence that good barn ventilation is a protective factor in this regard. In our study, caregivers of racehorses had an increased prevalence of MRS colonization compared with caregivers of show or pleasure horses. Whether this is associated with negative health outcomes in this occupational niche remains undetermined. Finally, barn-level horse-administered antimicrobial treatment was a significant risk factor for human MRS colonization. These findings emphasize the importance of antimicrobial resistance surveillance programs based on One Health approaches in workplaces where human‒animal contact occurs on a regular basis.

## Supplementary Information

**Additional file 1. Supplementary methods and results**.

**Additional file 2. Questionnaires used for collecting barn, horse, and personnel data**.

**Additional file 3. List of antimicrobial molecules tested for residues in feed**.

## Data Availability

The datasets used and/or analysed during the current study are available from the corresponding author upon reasonable request.

## References

[1] Hogan PG, Mork RL, Boyle MG, Muenks CE, Morelli JJ, Thompson RM, Sullivan ML, Gehlert SJ, Merlo JR, McKenzie MG, Wardenburg JB, Rzhetsky A, Burnham CD, Fritz SA (2019) Interplay of personal, pet, and environmental colonization in households affected by community-associated methicillin-resistant *Staphylococcus aureus*. J Infect 78:200–207 30503843 10.1016/j.jinf.2018.11.006 PMC6408279

[2] Zainab SM, Junaid M, Xu N, Malik RN (2020) Antibiotics and antibiotic resistant genes (ARGs) in groundwater: a global review on dissemination, sources, interactions, environmental and human health risks. Water Res 187:116455 33032106 10.1016/j.watres.2020.116455

[3] Grundmann H, Aires-de-Sousa M, Boyce J, Tiemersma E (2006) Emergence and resurgence of meticillin-resistant *Staphylococcus aureus* as a public-health threat. Lancet 368:874–885 16950365 10.1016/S0140-6736(06)68853-3

[4] Bollig ER, Granick JL, Webb TL, Ward C, Beaudoin AL (2022) A quarterly survey of antibiotic prescribing in small animal and equine practices-Minnesota and North Dakota, 2020. Zoonoses Public Health 69:864–874 35643964 10.1111/zph.12979 PMC9796041

[5] Fitzgerald JR (2012) Livestock-associated *Staphylococcus aureus*: origin, evolution and public health threat. Trends Microbiol 20:192–198 22386364 10.1016/j.tim.2012.01.006

[6] Mehta A, Rodrigues C, Kumar R, Rattan A, Sridhar H, Mattoo V, Ginde V (1996) A pilot programme of MRSA surveillance in India. (MRSA Surveillance Study Group). J Postgrad Med 42:1–3 9715287

[7] Van Embden JD, Van Soolingen D, Heersma HF, De Neeling AJ, Jones ME, Steiert M, Grek V, Mooi FR, Verhoef J (1996) Establishment of a European network for the surveillance of *Mycobacterium tuberculosis*, MRSA and penicillin-resistant pneumococci. J Antimicrob Chemother 38:905–907 8961063 10.1093/jac/38.5.905

[8] Johnson AP, Davies J, Guy R, Abernethy J, Sheridan E, Pearson A, Duckworth G (2012) Mandatory surveillance of methicillin-resistant *Staphylococcus aureus* (MRSA) bacteraemia in England: the first 10 years. J Antimicrob Chemother 67:802–809 22223229 10.1093/jac/dkr561

[9] Schonfeld V, Diercke M, Gilsdorf A, Eckmanns T, Walter J (2018) Evaluation of the statutory surveillance system for invasive MRSA infections in Germany, 2016–2017. BMC Public Health 18:1063 30143016 10.1186/s12889-018-5971-y PMC6109305

[10] Jordan D, Simon J, Fury S, Moss S, Giffard P, Maiwald M, Southwell P, Barton MD, Axon JE, Morris SG, Trott DJ (2011) Carriage of methicillin-resistant *Staphylococcus aureus* by veterinarians in Australia. Aust Vet J 89:152–159 21495985 10.1111/j.1751-0813.2011.00710.x

[11] Becker K, Both A, Weisselberg S, Heilmann C, Rohde H (2020) Emergence of coagulase-negative staphylococci. Expert Rev Anti Infect Ther 18:349–366 32056452 10.1080/14787210.2020.1730813

[12] Becker K, Heilmann C, Peters G (2014) Coagulase-negative staphylococci. Clin Microbiol Rev 27:870–926 25278577 10.1128/CMR.00109-13 PMC4187637

[13] Catry B, Van Duijkeren E, Pomba MC, Greko C, Moreno MA, Pyörälä S, Ruzauskas M, Sanders P, Threlfall EJ, Ungemach F, Törneke K, Munoz-Madero C, Torren-Edo J (2010) Reflection paper on MRSA in food-producing and companion animals: epidemiology and control options for human and animal health. Epidemiol Infect 138:626–644 20141646 10.1017/S0950268810000014

[14] Loeffler A, Pfeiffer DU, Lindsay JA, Soares Magalhaes RJ, Lloyd DH (2011) Prevalence of and risk factors for MRSA carriage in companion animals: a survey of dogs, cats and horses. Epidemiol Infect 139:1019–1028 20943000 10.1017/S095026881000227X

[15] Weese JS, Lefebvre SL (2007) Risk factors for methicillin-resistant *Staphylococcus aureus* colonization in horses admitted to a veterinary teaching hospital. Can Vet J 48:921–926 17966332 PMC1950112

[16] Weese JS, Rousseau J, Traub-Dargatz JL, Willey BM, McGeer AJ, Low DE (2005) Community-associated methicillin-resistant *Staphylococcus aureus* in horses and humans who work with horses. J Am Vet Med Assoc 226:580–583 15742700 10.2460/javma.2005.226.580

[17] Adams R, Smith J, Locke S, Phillips E, Erol E, Carter C, Odoi A (2018) An epidemiologic study of antimicrobial resistance of *Staphylococcus* species isolated from equine samples submitted to a diagnostic laboratory. BMC Vet Res 14:42 29402294 10.1186/s12917-018-1367-6 PMC5800099

[18] EFSA Panel on Animal Health and Welfare (AHAW), Nielsen SS, Bicout DJ, Calistri P, Canali E, Drewe JA, Garin-Bastuji B, Gonzales Rojas JL, Gortazar Schmidt C, Herskin M, Michel V, Miranda Chueca MA, Padalino B, Pasquali P, Roberts HC, Sihvonen LH, Spoolder H, Stahl K, Velarde A, Viltrop A, Winckler C, Dewulf J, Guardabassi L, Hilbert F, Mader R, Baldinelli F, Alvarez J (2021) Assessment of animal diseases caused by bacteria resistant to antimicrobials: horses. EFSA J 19:e0711234987627

[19] Roudaud M, Allano M, Fairbrother JH, Sauve F (2020) A retrospective study on methicillin-resistant Staphylococcus spp. isolated from horses admitted to a Canadian veterinary teaching hospital between 2008 and 2018. Can Vet J 61:1197–1202 33149358 PMC7560767

[20] Tirosh-Levy S, Steinman A, Carmeli Y, Klement E, Navon-Venezia S (2015) Prevalence and risk factors for colonization with methicillin resistant *Staphylococcus aureus* and other Staphylococci species in hospitalized and farm horses in Israel. Prev Vet Med 122:135–144 26417658 10.1016/j.prevetmed.2015.09.007

[21] Mallardo K, Nizza S, Fiorito F, Pagnini U, De Martino L (2013) A comparative evaluation of methicillin-resistant staphylococci isolated from harness racing-horses, breeding mares and riding-horses in Italy. Asian Pac J Trop Biomed 3:169–173 23620832 10.1016/S2221-1691(13)60044-1 PMC3631744

[22] Peterson AE, Davis MF, Awantang G, Limbago B, Fosheim GE, Silbergeld EK (2012) Correlation between animal nasal carriage and environmental methicillin-resistant *Staphylococcus aureus* isolates at U.S. horse and cattle farms. Vet Microbiol 160:539–543 22795260 10.1016/j.vetmic.2012.06.032

[23] Van den Eede A, Martens A, Feryn I, Vanderhaeghen W, Lipinska U, Gasthuys F, Butaye P, Haesebrouck F, Hermans K (2012) Low MRSA prevalence in horses at farm level. BMC Vet Res 8:213 23134703 10.1186/1746-6148-8-213 PMC3536571

[24] Groves MD, Crouch B, Coombs GW, Jordan D, Pang S, Barton MD, Giffard P, Abraham S, Trott DJ (2016) Molecular epidemiology of methicillin-resistant *Staphylococcus aureus* isolated from Australian veterinarians. PLoS One 11:e0146034 26735694 10.1371/journal.pone.0146034 PMC4703204

[25] Moodley A, Nightingale EC, Stegger M, Nielsen SS, Skov RL, Guardabassi L (2008) High risk for nasal carriage of methicillin-resistant *Staphylococcus aureus* among Danish veterinary practitioners. Scand J Work Environ Health 34:151–157 18470441 10.5271/sjweh.1219

[26] Maddox TW, Pinchbeck GL, Clegg PD, Wedley AL, Dawson S, Williams NJ (2012) Cross-sectional study of antimicrobial-resistant bacteria in horses. Part 2: risk factors for faecal carriage of antimicrobial-resistant *Escherichia coli* in horses. Equine Vet J 44:297–303 21848536 10.1111/j.2042-3306.2011.00440.x

[27] Van den Eede A, Hermans K, Van den Abeele A, Flore K, Dewulf J, Vanderhaeghen W, Némeghaire S, Butaye P, Gasthuys F, Haesebrouck F, Martens A (2013) The nasal vestibulum is the optimal sampling site for MRSA screening in hospitalised horses. Vet J 197:415–419 23465751 10.1016/j.tvjl.2013.01.031

[28] Bocher S, Smyth R, Kahlmeter G, Kerremans J, Vos MC, Skov R (2008) Evaluation of four selective agars and two enrichment broths in screening for methicillin-resistant *Staphylococcus aureus*. J Clin Microbiol 46:3136–3138 18632905 10.1128/JCM.00478-08 PMC2546728

[29] Smyth RW, Kahlmeter G (2005) Mannitol salt agar-cefoxitin combination as a screening medium for methicillin-resistant *Staphylococcus aureus*. J Clin Microbiol 43:3797–3799 16081913 10.1128/JCM.43.8.3797-3799.2005 PMC1233994

[30] Stegger M, Andersen PS, Kearns A, Pichon B, Holmes MA, Edwards G, Laurent F, Teale C, Skov R, Larsen AR (2012) Rapid detection, differentiation and typing of methicillin-resistant *Staphylococcus aureus* harbouring either mecA or the new mecA homologue mecA(LGA251). Clin Microbiol Infect 18:395–400 22429460 10.1111/j.1469-0691.2011.03715.x

[31] Johnston R, Jones K, Manley D (2018) Confounding and collinearity in regression analysis: a cautionary tale and an alternative procedure, illustrated by studies of British voting behaviour. Qual Quant 52:1957–1976 29937587 10.1007/s11135-017-0584-6 PMC5993839

[32] Boyle AG, Timoney JF, Newton JR, Hines MT, Waller AS, Buchanan BR (2018) *Streptococcus equi* infections in horses: guidelines for treatment, control, and prevention of strangles-revised consensus statement. J Vet Intern Med 32:633–647 29424487 10.1111/jvim.15043 PMC5867011

[33] Dayaram A, Seeber PA, Greenwood AD (2021) Environmental detection and potential transmission of equine herpesviruses. Pathogens 10:423 33916280 10.3390/pathogens10040423 PMC8066653

[34] Busscher JF, van Duijkeren E, van Sloet MM (2006) The prevalence of methicillin-resistant staphylococci in healthy horses in the Netherlands. Vet Microbiol 113:131–136 16303264 10.1016/j.vetmic.2005.10.028

[35] Maddox TW, Clegg PD, Diggle PJ, Wedley AL, Dawson S, Pinchbeck GL, Williams NJ (2012) Cross-sectional study of antimicrobial-resistant bacteria in horses. Part 1: prevalence of antimicrobial-resistant *Escherichia coli* and methicillin-resistant *Staphylococcus aureus*. Equine Vet J 44:289–296 21848534 10.1111/j.2042-3306.2011.00441.x

[36] Bortolami A, Williams NJ, McGowan CM, Kelly PG, Archer DC, Corro M, Pinchbeck G, Saunders CJ, Timofte D (2017) Environmental surveillance identifies multiple introductions of MRSA CC398 in an Equine Veterinary Hospital in the UK, 2011–2016. Sci Rep 7:5499 28710350 10.1038/s41598-017-05559-8 PMC5511188

[37] Carfora V, Caprioli A, Grossi I, Pepe M, Alba P, Lorenzetti S, Amoruso R, Sorbara L, Franco A, Battisti A (2016) A methicillin-resistant *Staphylococcus aureus* (MRSA) sequence type 8, spa type t11469 causing infection and colonizing horses in Italy. Pathog Dis 74:ftw025 27052029 10.1093/femspd/ftw025

[38] Cuny C, Abdelbary MMH, Kock R, Layer F, Scheidemann W, Werner G, Witte W (2015) Methicillin-resistant *Staphylococcus aureus* from infections in horses in Germany are frequent colonizers of veterinarians but rare among MRSA from infections in humans. One Health 2:11–17 28616471 10.1016/j.onehlt.2015.11.004 PMC5441336

[39] Van den Eede A, Martens A, Flore K, Denis O, Gasthuys F, Haesebrouck F, Van den Abeele A, Hermans K (2013) MRSA carriage in the equine community: an investigation of horse-caretaker couples. Vet Microbiol 163:313–318 23434186 10.1016/j.vetmic.2012.12.038

[40] Loncaric I, Tichy A, Handler S, Szostak MP, Tickert M, Diab-Elschahawi M, Spergser J, Künzel F (2019) Prevalence of methicillin-resistant *Staphylococcus* sp. (MRS) in different companion animals and determination of risk factors for colonization with MRS. Antibiotics 8:36 30959767 10.3390/antibiotics8020036 PMC6627599

[41] Rossi TM, Moore A, O’Sullivan TL, Greer AL (2019) Equine rhinitis a virus infection at a standardbred training facility: incidence, clinical signs, and risk factors for clinical disease. Front Vet Sci 6:71 30918893 10.3389/fvets.2019.00071 PMC6424864

[42] Weese JS, Sabino C (2005) Scrutiny of antimicrobial use in racing horses with allergic small airway inflammatory disease. Can Vet J 46:438–489 16018565 PMC1090451

[43] Hobday RA, Dancer SJ (2013) Roles of sunlight and natural ventilation for controlling infection: historical and current perspectives. J Hosp Infect 84:271–282 23790506 10.1016/j.jhin.2013.04.011 PMC7132476

[44] Angen O, Nielsen MW, Lofstrom P, Larsen AR, Hendriksen NB (2021) Airborne spread of methicillin resistant *Staphylococcus aureus* from a swine farm. Front Vet Sci 8:644729 34150881 10.3389/fvets.2021.644729 PMC8211894

[45] Bai H, He LY, Wu DL, Gao FZ, Zhang M, Zou HY, Yao MS, Ying GG (2021) Spread of airborne antibiotic resistance from animal farms to the environment: dispersal pattern and exposure risk. Environ Int 158:106927 34673316 10.1016/j.envint.2021.106927

[46] Bos ME, Verstappen KM, van Cleef BA, Dohmen W, Dorado-Garcia A, Graveland H, Duim B, Wagenaar JA, Kluytmans JA, Heederik DJ (2016) Transmission through air as a possible route of exposure for MRSA. J Expo Sci Environ Epidemiol 26:263–269 25515375 10.1038/jes.2014.85

[47] Kaiser-Thom S, Gerber V, Collaud A, Hurni J, Perreten V (2022) Prevalence and WGS-based characteristics of *Staphylococcus aureus* in the nasal mucosa and pastern of horses with equine pastern dermatitis. BMC Vet Res 18:79 35209904 10.1186/s12917-021-03053-y PMC8867626

[48] Congdon ST, Guaglione JA, Ricketts OMA, Murphy KV, Anderson MG, Trowbridge DA, Al-Abduladheem Y, Phillips AM, Beausoleil AM, Stanley AJ, Becker TJ, Silver AC (2023) Prevalence and antibiotic resistance of *Staphylococcus aureus* associated with a college-aged cohort: life-style factors that contribute to nasal carriage. Front Cell Infect Microbiol 13:1195758 37441241 10.3389/fcimb.2023.1195758 PMC10333693

[49] Liu G, Thomsen LE, Olsen JE (2022) Antimicrobial-induced horizontal transfer of antimicrobial resistance genes in bacteria: a mini-review. J Antimicrob Chemother 77:556–567 34894259 10.1093/jac/dkab450

[50] Martins A, Cunha Mde L (2007) Methicillin resistance in *Staphylococcus aureus* and coagulase-negative staphylococci: epidemiological and molecular aspects. Microbiol Immunol 51:787–795 17895595 10.1111/j.1348-0421.2007.tb03968.x

[51] EFSA Panel on Biological Hazards (BIOHAZ), Koutsoumanis K, Allende A, Alvarez-Ordóñez A, Bolton D, Bover-Cid S, Chemaly M, Davies R, De Cesare A, Herman L, Hilbert F, Lindqvist R, Nauta M, Ru G, Simmons M, Skandamis P, Suffredini E, Andersson DI, Bampidis V, Bengtsson-Palme J, Bouchard D, Ferran A, Kouba M, López Puente S, López-Alonso M, Nielsen SS, Pechová A, Petkova M, Girault S, Broglia A, Guerra B, Innocenti ML, Liébana E, López-Gálvez G, Manini P, Stella P, Peixe L (2021) Maximum levels of cross-contamination for 24 antimicrobial active substances in non-target feed Part 13: Diaminopyrimidines: trimethoprim. EFSA J 19:e06865 34729093 10.2903/j.efsa.2021.6865 PMC8546793

[52] EFSA Panel on Biological Hazards (BIOHAZ), Koutsoumanis K, Allende A, Alvarez-Ordóñez A, Bolton D, Bover-Cid S, Chemaly M, Davies R, De Cesare A, Herman L, Hilbert F, Lindqvist R, Nauta M, Ru G, Simmons M, Skandamis P, Suffredini E, Andersson DI, Bampidis V, Bengtsson-Palme J, Bouchard D, Ferran A, Kouba M, López Puente S, López-Alonso M, Nielsen SS, Pechová A, Petkova M, Girault S, Broglia A, Guerra B, Innocenti ML, Liébana E, López-Gálvez G, Manini P, Stella P, Peixe L (2021) Maximum levels of cross-contamination for 24 antimicrobial active substances in non-target feed. Part 4: beta-lactams: Amoxicillin and penicillin V. EFSA J 19:e0685534729084

[53] Avolio R, Pederiva S, Morello S, Crescio MI, Ru G, Grifoni F, Abete MC, Marchis D (2021) Official controls on carry-over of antibiotics in feed: a useful tool to contain the development of antibiotic resistance. Anim Husb Dairy Vet Sci 5:1–3 10.15761/AHDVS.1000185

[54] European Medicines Agency ESoVAC (2022) Sales of veterinary antimicrobial agents in 31 European countries in 2022. (EMA/299538/2023) https://www.ema.europa.eu/en/documents/report/sales-veterinary-antimicrobial-agents-31-european-countries-2022-trends-2010-2022-thirteenth-esvac-report_en.pdf. Accessed 18 July 2024

