# Supplementary material for: Prevalence and risk factors associated with nasal carriage of methicillin-resistant staphylococci in horses and their caregivers
Source: Vet Res. 2024 Sep 9;55:108. doi: 10.1186/s13567-024-01364-0 (PMC11386249; doi:10.1186/s13567-024-01364-0)
Supplement: Supplementary file 1 — Additional file 1. Supplementary methods and results. [file 13567_2024_1364_MOESM1_ESM.docx]

**Additional file 1 Supplementary methods and results**

**Methods**

*Data collection*

Barn-level data were collected by interviewing the owner(s) and/or personnel: main activity of the horses stabled at the barn (racing, show riding, pleasure riding), total number of horses stabled, number of attending veterinarians following the horses stabled within the barn, estimated number of antimicrobial therapies (prescriptions) administered to all the horses stabled at the barn during the last 12 months (used to build the variables labelled as “barn-estimated” antimicrobial treatment), and whether every horse had its own halter and/or harness or not. Whenever possible, barn-level questions were also asked to horse owners or to barn’s personnel not included in the study to validate the responses. In case of contrasting information, data were excluded from the analysis.

Horse-level data collected were: age, sex, weight, breed, intended use (human consumption or not), time in months spent at the barn since its arrival (length of stay or persistence time), presence or absence of windows in the box (in addition to the door which, itself, often has a window; minimal surface 80 × 80 cm^2^), amount of time spent in paddock per day (hours), and number of transportations during the last two months. Also, data concerning previous hospitalizations and/or antimicrobial treatments administered in the last 12 months were obtained. The latter was used to build the variables defined as “horse-specific antimicrobial treatment”. Whenever possible, data concerning pharmacological treatments were validated by interviewing the attending veterinarian.

Personnel-level data collected were: age, sex, height, health status (healthy vs. presenting at least one disease, either acute or chronic), assumption of antimicrobial in the last 2 months, time in months spent at the barn since his/her first hiring, number of horses he/she cares for daily at the barns, time spent daily at the barn (hours), time spent daily at the barn in direct contact with horses (hours), time spent daily in other barns (hours), handwashing habits while at the barns (daily frequency).

*Sampling*

The swabs were moistened with few drops of sterile saline using a syringe before being inserted in the left and then in the right nostril of the horse for approximately 15 cm and rubbed gently against the mucosa for 5 seconds. While withdrawing the swab, care was taken to also sample the region of the nasal diverticulum {Van den Eede, 2013 #5572}. Contaminated swabs by contact with the operator or the horse’s skin during the procedure were discarded. The environmental sample was collected over a standard area of 10 cm^2^ delimited by a plastic mask by firm rubbing of the swab with rotation for about 10 s. The environmental sample was collected at similar locations for every barn, namely the wall of the box or area used for showering the horses. The plastic mask was cleaned with water and alcohol 5 min before and after every use.

*MRS isolation and identification*

Pre-enrichment was implemented by incubating the swabs in 5 mL of Mueller Hinton Broth, 6% NaCl for 10 min to improve the sensitivity of the protocol to detect MRS spp. [17]. The broth was incubated at 37 ± 1 °C for 18-24 h. Then, 1 mL of MHB-salt was added to 9 mL di Tryptone Soy Broth with 20 mg/L aztreonam, 3.5 mg/L cefoxitine and further incubated at 37 ± 1 °C for 18-24 h [17, 18]. Broth was plated on Mannitol Salt Agar with 6% NaCl with cefoxitine 3.5 mg/L. The plates were incubated at 37 ± 1 °C for 24-48 h. Isolated colonies were then plated on Columbia blood agar and incubated at 37 ± 1 °C for 24-48 h. Suspected colonies of MRS spp. were identified using standard techniques: colony morphology, gram staining, culture media colour changes, catalase and coagulase tests. Bacterial species identification was performed by means of whole cell MALDI-TOF mass spectrometry. DNA was extracted from the isolates and PCR was performed for 16S, mecA, mecC and nuc genes as previously described [19].

*Antimicrobial susceptibility testing*

The European Committee on Antimicrobial Susceptibility Testing (EUCAST 2019) breakpoints were used to define resistance (intermediate were considered as resistant). As EUCAST breakpoints are not available for ceftiofur Na and enrofloxacin, the Clinical and Laboratory Standards Institute (CLSI 2019) breakpoints were used instead. Staphylococci spp. resistant to 3 or more antimicrobial molecules among those tested were defined as multi-drug resistant Staphylococci (MDRS).

*Statistical analysis*

The confidence intervals (CI) of prevalence and prevalence ratios (PR) for MRS and MDRS carriage were calculated using a robust variance estimate that adjusts for within-cluster correlation and negates the need for independent observations, requiring only that clusters are independent. The frequency of travelling during the last 2 months and of antimicrobial treatments during the last 12 months were calculated as rates, dividing the sum of the events (travel or antimicrobial treatments) by horse months at risk during the considered period. For barn-estimated data, time at risk for the horses non studied was considered to be 6 months (over 1 year observation period), as we did ignore the moment of arrival of those horses at the barn. Travelling rate ratio and antimicrobial treatment rate ratio were obtained using Poisson regression with robust cluster variance estimates.

**Results**

*Details on non-Staphylococcal methicillin-resistant isolates*

A total of 51/110 (46%) horses harboured 63 isolates that showed phenotypical resistance against methicillin. Among bacterial isolates from equine samples, after species identification, 34 isolates from 33 horses were identified as MRS. One of them (a strain of *S. sciuri* not expressing *mecA* isolated from a Standardbred horse from which a MDR *S. lentus* was also yielded) was sensible to all the antimicrobial molecules tested, including penicillin and ceftiofur. It was considered as a sensible species whose growth was facilitated by the close relationship with the MRD *S. lentus*, and discarded from further analyses. This yielded a total of 33 MRS isolates from 33 horses, corresponding to an overall 30% prevalence of healthy horses with nasal colonization by MRS in our region (95% CI 20.7-41.3%). The remaining non-MRS isolates were identified as *Enterococcus spp.* (*n* = 14), *Serratia spp.* (*n* = 9), mixed bacterial flora (*n* = 3), or identification yielded no match (*n* = 3).

As for human samples, A total of 15 methicillin-resistant isolates were identified in horse caregivers, of which 11 were classified as MRS. The 4 non-*Staphylococcus spp.* methicillin-resistant isolates were identified from 3 people, consisting in 3 *Enterococcus spp.* and 1 *Serratia spp.* strains.

**Additional file 1A** **Bivariate unadjusted Poisson regression analysis with cluster variance (robust).** Results expressed as PR (prevalence ratio) for a horse of being a nasal carrier of ≥1 MRS. *n* = 110.

|  | PR | 90% CI | |
| --- | --- | --- | --- |
| Age [years] | 0.97 | 0.93 | 1.01 |
| Sex [ref: female] | 0.68 | 0.42 | 1.10 |
| Weight [kg] | 1.00 | 1.00 | 1.00 |
| **Barn activity** |  |  |  |
| Pleasure riding | ref | - | - |
| Show riding | 1.14 | 0.59 | 2.20 |
| **Racing** | **1.84** | **1.02** | **3.31** |
| Number of horses stabled at the barn |  |  |  |
| ≤20 horses | ref | - | - |
| >20 and ≤40 horses | 1.00 | 0.61 | 1.63 |
| >40 horses | 0.48 | 0.15 | 1.48 |
| Number of vets operating at the barn |  |  |  |
| 1 vet | ref | - | - |
| >1 vet | 1.39 | 0.86 | 2.25 |
| Environmental colonization by MRS [yes/no] | 0.69 | 0.28 | 1.65 |
| **Shared tack [yes/no]** | **1.96** | **1.21** | **3.19** |
| **Barn-estimated antimicrobial treatment ratio** | **1.67** | **1.20** | **2.32** |
| Horse-specific antimicrobial treatment ratio | 0.93 | 0.39 | 2.21 |
| Intended for food production [yes/no; ref: no] | 1.12 | 0.48 | 2.60 |
| Time at the barn [months] | 1.00 | 1.00 | 1.00 |
| **Box with >1 windows [yes/no; ref: no]** | **0.52** | **0.31** | **0.85** |
| Paddock [yes/no; ref: no] | 0.92 | 0.53 | 1.60 |
| Paddock [hours/day] | 0.99 | 0.96 | 1.01 |
| **Ventilation index** |  |  |  |
| Score 0 | ref | - | - |
| **Score 1** | **0.38** | **0.21** | **0.69** |
| **Score 2** | **0.40** | **0.20** | **0.79** |
| Travel in the last 2 months [n] | 0.97 | 0.76 | 1.28 |
| Hospitalization in the last 12 months [yes/no; ref: no] | 1.39 | 0.54 | 3.57 |
| Antimicrobial treatment in the last 12 months [yes/no; ref: no] | 1.06 | 0.71 | 1.60 |

Bold indicates variables significantly associated with MRS carriage in horses studied.

**Additional file 1B** **Bivariate unadjusted Poisson regression analysis with cluster variance (robust).** Results are expressed as the prevalence ratios (PR) and estimate the association of the variables listed with the status of human nasal carrier of one or more MRS. *n* = 34.

|  | PR | 90% CI | |
| --- | --- | --- | --- |
| Age [years] | 1.02 | 0.99 | 1.06 |
| **Sex [ref: female]** | **3.55** | **1.10** | **11.45** |
| Height [cm] | 1.01 | 0.96 | 1.06 |
| **Barn activity** |  |  |  |
| Pleasure riding | ref | - | - |
| Show riding | 0.65 | 0.09 | 4.43 |
| **Racehorses** | **4.73** | **1.53** | **14.62** |
| Working experience [years] | 1.00 | 0.96 | 1.05 |
| **Barn-estimated antimicrobial treatment ratio** | **3.32** | **1.95** | **5.67** |
| Chronic disease [yes/no; ref: no] | 0.75 | 0.17 | 3.39 |
| N horses stabled at the barn | 0.98 | 0.96 | 1.01 |
| **N horses carrying MRS** | **1.71** | **1.15** | **2.55** |
| N horses manipulated daily at the barn | 0.98 | 0.96 | 1.01 |
| Daily time spent at the barn [hours] | 0.99 | 0.91 | 1.07 |
| Daily time spent in close contact with horses [hours] | 1.04 | 0.92 | 1.17 |
| N horses manipulated daily outside the barn | 0.85 | 0.69 | 1.04 |
| Daily frequency hand washing | 0.98 | 0.91 | 1.04 |
| **Mate caregiver carrying intranasal MRS** | **3.33** | **1.53** | **7.24** |

Bold indicates variables significantly associated with MRS carriage.
