# Supplementary material for: Prevalence and risk factors associated with nasal carriage of methicillin-resistant staphylococci in horses and their caregivers
Source: Vet Res. 2024 Sep 9;55:108. doi: 10.1186/s13567-024-01364-0 (PMC11386249; doi:10.1186/s13567-024-01364-0)
Supplement: Supplementary file 2 — Additional file 2. Questionnaires used for collecting barn, horse, and personnel data. [file 13567_2024_1364_MOESM2_ESM.docx]

**Supplementary item 2. Questionnaires used for collecting barn, horse, and personnel data.**

(A) Questionnaire used for barn data.

BARN DATA:

Name of the barn: …………………………………………………………………………………..

Address ……………………………………………………………………………………………..

Main barn activity: ………………………………………………………………………………….

Number of horses stabled: ………………………………………………………………………….

Number of vets operating in the barn: ……………………………………………………………...

Estimate N antimicrobial treatments performed at the barn in the last 12 months: ……………….

Anonymous ID of the barn : S-…….

(B) Questionnaire used for personnel data.

PERSONNEL DATA:

Anonymous ID of the person: P-………

Anonymous ID of the barn: S-………

For how long you have been working at this barn (months)? ............................................................

Sex: M F

Age: …………………………………………………………………………………………………..

Any disease? …………………………………………………………………………………………

Any antimicrobial treatment in the last 2 months? Y N

If YES, which ones? ………………………………………………………………………………….

Heigh in cm: ………………………………………………………………………………………..…

With how many horses from this barn you are in contact with every day? ……………………..……

How many hours per day do you spend at this barn? …………………………………………..……..

How many hours per day do you spend at this barn in direct contact (manipulation) with horses? .....

With how many horses from others barn you are in contact with every day? ………………..……...

If pertinent, how many hours per day do you spend in other barns? ..................................................

If pertinent, how many of them in direct contact with horses from other barns? …………................

In this barn, are tacks shared among horses? ......................................................................................

How many times per day do you wash your hands? ………………………………….………………

(C) Questionnaire used for horses’ data.

HORSE DATA:

Anonymous ID of the horse: C-…………

Anonymous ID of the barn: S-…………….

Sex: M F

Age (years): ……………… Weight estim (kg): …………………….

Breed: ………………………………………………………………………………………………..

Main activity: ………………………………………………………………………………………..

Intended for food production? Y N

For how long the horse has been at the barn? ……………………………………………………….

Does the box of the horse has >1 window? Y N N/A

Does the horse spend time at the paddock? Y N

If YES, how many hours per day? ……………………………………………….…………………..

N of transportation the horse had during the last 2 months: …………………….….………………..

How many times the horse has been visited by a vet during the last 12 months and for which reason(s)? ……………………………………………………………………….................................................

Has the horse been to the hospital during the last 12 months? Y N

Which drugs has the horse been administered during the last 12 months? …………………………………………………………………………………………………………

Did the horse receive any antimicrobial treatment during the last 12 months? Y N

If YES, which one(s)? Specify molecule and treatment regimen ………………………………………………………………………………………………………...

If YES, for what reason was the antimicrobial treatment administered? …………………………….

If YES, did the horse respond to the treatment? …..…………………………………………………
