# Supplementary material for: Prevalence and risk factors associated with nasal carriage of methicillin-resistant staphylococci in horses and their caregivers
Source: Vet Res. 2024 Sep 9;55:108. doi: 10.1186/s13567-024-01364-0 (PMC11386249; doi:10.1186/s13567-024-01364-0)
Supplement: Supplementary file 3 — Additional file 3. List of antimicrobial molecules tested for residues in feed. [file 13567_2024_1364_MOESM3_ESM.docx]

**Additional file 3.** **List of the antimicrobial molecules tested for residues in feed.**

| **CLASS** | **MOLECULE** |
| --- | --- |
| **AMPHENICOLS** | CLORAMPHENICOL |
|  | FLORPHENICOL |
|  | TIAMPHENICOL |
| **QUINOLONES** | NALIDIXIC ACID |
|  | OXOLINIC ACID |
|  | CIPROFLOXACIN |
|  | DANOFLOXACIN |
|  | DIFLOXACIN |
|  | ENROFLOXACIN |
|  | FLUMEQUIN |
|  | LEVOFLOXACIN |
|  | MARBOFLOXACIN |
|  | NORFLOXACIN |
|  | SARAFLOXACIN |
| **DIAMINOPYRIMIDINE** | TRIMETOPRIM |
| **LINCOSAMIDIES** | LINCOMYCIN |
| **MACROLIDES** | ERITROMYCIN A |
|  | GAMITROMYCIN |
|  | JOSAMYCIN |
|  | KITASAMYCIN |
|  | NEOSPIRAMICIN I |
|  | OLEANDOMYCIN |
|  | SPIRAMYCIN |
|  | TILMICOSIN |
|  | TILOSIN |
| **PENICILLINS** | AMOXICILLIN |
|  | AMPICILLIN |
| **PLEUROMUTILINS** | TIAMULIN |
| **POLIPEPTIDES** | VIRGINAMYCIN S1 |
| **SULFONAMIDES** | SULFACHINOXALIN |
|  | SULFADIAZINE |
|  | SULFADIMETOXINE |
|  | SULFAMERAZINE |
|  | SULFAMETAZINE |
|  | SULFAMETOXAZOLE |
|  | SULFAMETOXIPIRIDAZINE |
|  | SULFAMONOMETOXINE |
|  | SULFAPIRIDINE |
|  | SULFATIAZOLE |
| **TETRACYCLINES** | CHLORTETRACYCLINE |
|  | DOXYCYCLINE |
|  | OXITETRACYCILINE |
|  | TETRACYCLINE |
